# Buffering – Please Be Patient! Does the Attribution of Pauses to Technical Issues Hamper Learning?

**DOI:** 10.3389/fpsyg.2021.771394

**Published:** 2021-10-22

**Authors:** Martin Merkt

**Affiliations:** German Institute for Adult Education – Leibniz Centre for Lifelong Learning, Bonn, Germany

**Keywords:** video-based learning, pauses, technical issues, didactical pauses, online learning

## Abstract

In educational contexts, system-determined pauses are often used to interrupt the transient flow of information and thus avoid cognitive overload in dynamic learning materials. However, next to these didactically motivated interruptions, video-based learning materials may also be interrupted due to technical issues with regard to constrained bandwidth or outdated technology. Against this background, the current experiment investigated whether the interruption of dynamic representations due to technical issues negatively affects learning. For this purpose, 64 participants watched an Arabic language tutorial. They were either informed that the video included breaks in order to support learning or that there may be breaks due to technical issues. Contrary to our pre-registered hypotheses, the attribution of the pauses to technical issues did not hamper learning and did not affect participants' ratings regarding the usefulness and the disturbance caused by the pauses. However, exploratory analyses revealed a negative correlation between the perceived usefulness and the disturbance caused by the pauses. Limitations and implications of these findings are discussed.

## Introduction

The Corona pandemic both fueled the popularity of videos as an educational resource (Skulmowski and Rey, 2020) and unveiled deficits in the technological infrastructure in rural areas (Amankwah-Amoah et al., 2021; Faraj et al., 2021). In this manuscript, we combine both of these recent developments by investigating whether the attribution of pauses in videos either to a didactical purpose or to technical issues differentially affects learners' perception of the pauses as useful or disturbing and thus has implications for the learning outcomes.

To the best of our knowledge, in none of the studies investigating the effects of system-determined pauses on learning, the interruptions were attributed to technical issues. In contrast, the effect of interrupting the transient flow of information in dynamic learning materials such as videos and animations has to date only been investigated with system-determined pauses that were included to facilitate the learning process (e.g., Hasler et al., 2007; Spanjers et al., 2011; Merkt et al., 2018). In particular, even though videos are generally considered to be a popular medium which is erroneously assumed to convey information in a simple to understand fashion (see Salomon, 1984; Kardas and O'Brien, 2018), videos task learners with continuously paying attention to the learning materials in order not to miss relevant information (transient information effect, Leahy and Sweller, 2011).

In the framework of the cognitive load theory (Sweller et al., 1998, 2019), transient information may result in cognitive overload because it may exhaust the learners' limited cognitive resources. Addressing this issue, instructional designers often provide learners with system-determined pauses. The beneficial effect of such system-determined pauses in dynamic learning materials was frequently demonstrated (Hasler et al., 2007; Rey et al., 2019), especially for participants who are at risk for cognitive overload (Lusk et al., 2009; Spanjers et al., 2011). Further, Lee et al. (2020) observed that learners' cognitive load was reduced during user-determined pauses in a medical simulation game. The authors attributed this as support for the assumption that pauses help prevent cognitive overload.

Next to the prevention of cognitive overload, it may be argued that the introduction of pauses in videos is most beneficial if learners use the pauses to actively elaborate the learning materials in a way that supports later retrieval of the information (see Cheon et al., 2014). Supporting this argument, Cheon et al. (2014) observed that learning outcomes were supported by instructing participants to perform a free recall task during system-determined pauses in a dynamic presentation. Whereas this study lends support to the assumption that encouraging elaboration processes during pauses supports learning, it has not yet been investigated whether the disruption of such elaboration processes (for example by attributing the pauses to technical issues instead of a didactical purpose) has a detrimental effect on learning. From an information processing perspective, interrupting the transient flow of information in dynamic representations should avoid cognitive overload independent of the attribution of the interruption to a didactical purpose or to a technical issue. However, it is feasible to assume that the learners' perception of these two different kinds of interruptions differs and thus differentially affects the learning outcomes. In this regard, technical difficulties have been identified as one of the major obstacles in online learning (Song et al., 2004; Kay, 2012). More specifically, technical challenges related to the use of video podcasts included file size and download time, which may both result in buffering (Kay, 2012).

Given the increasing popularity of online videos as an educational resource (Rat für kulturelle Bildung, 2019; Skulmowski and Rey, 2020), it is an important research question whether such buffering interruptions due to technical issues may have detrimental effects on the actual learning outcomes. To the best of our knowledge, this is the first experiment to test whether buffering due to technical issues in video-based learning materials has a negative effect on the learning outcomes. To keep learning time constant, the buffering condition was compared to a video including pauses that were purportedly introduced with a didactical purpose. Thus, the experiment is a strong test as to whether learning outcomes are affected by the mere attribution of the pauses to technical difficulties, while at the same time using a common measure to increase videos' effectiveness (i.e., didactical pauses) as a comparison condition. The following hypotheses were pre-registered on OSF^1^:

Hypothesis 1: Pauses that are attributed to the instructor's intention to facilitate the learning process (i.e., with a didactical purpose) result in better learning outcomes than pauses that are attributed to technical issues.
Hypothesis 2: Pauses that are attributed to technical issues are rated to be more disturbing than pauses that are attributed to a didactical purpose.
Hypothesis 3: The positions of the pauses are rated to be less useful if the pauses are attributed to technical issues than if the pauses are attributed to a didactical purpose.

## Method

### Participants

Overall, 75 participants took part in the experiment. After excluding ten participants who did not accurately remember the reasons for the interruptions (see Manipulation Check) and one participant who reported having previously participated in an Arabic language course^2^, 64 participants (49 female, 15 male) remained in the final sample. These participants' mean age was 20.91 years (SD = 2.85). Sixty-one participants reported German as their mother language, three participants spoke German for 8, 10, and 24 years respectively. No participant reported Arabic as his or her mother language. Participants were randomly assigned to one of two conditions resulting from the between subject variable attribution of pauses (technical issue vs. didactical purpose).

### Materials

#### Instructions

The attribution of the interruptions included in the video was manipulated with different instructions before the video started. In the didactical purpose condition, participants read the following information: “Now we show you a video in which you get a first introduction to the Arabic language. Please watch the video carefully to remember as many words as possible in a knowledge test. **To help you learn the information in the video, we will pause the video at various points for you**.” In the technical issue condition, participants read the following information: “Now we show you a video in which you get a first introduction to the Arabic language. Please watch the video carefully to remember as many words as possible in a knowledge test. **Unfortunately, due to the internet connection, the playback might pause at various points. We therefore ask for your patience**.” The respective attributions for the pauses were printed in boldface.

#### Video

The learning materials consisted of a video including narrated slides presenting the learners with basic vocabulary of the Arabic language. The narration was provided by a young female native speaker. The auditory narration was accompanied by the words in Latin writing and illustrating pictures representing the words. Overall, the video lasted for 815 seconds (including the pauses). There were 13 pauses in the video, with each pause lasting for 15 seconds. During the pauses, the picture track was not visible and an animated circular loading symbol was displayed. The loading symbol was displayed in both conditions to keep all factors beyond the attribution of pauses constant. The loading screen did not include any additional information such as text. The positions of the pauses were selected not to interrupt sentences or not to be within words. Participants in both conditions watched exactly the same video with the pauses presented at exactly the same positions. Participants could not engage in any interactions with the video (e.g., manual pauses).

### Measures

#### Prior Interest and Prior Knowledge

Prior interest and prior knowledge were measured with self-assessment questions. With regard to prior interest, we asked participants to indicate how much they were interested in learning languages in general (“How interested are you in learning foreign languages in general?”) and the Arabic language in specific (“How interested are you in learning the Arabic language?”). Both answers were recorded on scales ranging from 1 (not at all interested) to 7 (very interested). With regard to prior knowledge, we asked participants to rate how well they spoke (“How well do you speak the Arabic language?”) and understood (“How well do you understand the Arabic language?”) the Arabic language. Both answers were recorded on scales ranging from 1 (not good at all) to 7 (very good). It was also assessed whether participants had already taken part in an Arabic language course.

#### Knowledge Test

The knowledge test included 54 items. Participants were asked to give the German translations of 54 Arabic words printed in Latin writing. They were awarded one point for correct answers, 0.5 points for partially correct answers (e.g., naming the correct word with the inaccurate gender), and 0 points for incorrect answers, resulting in a maximum score of 54 points. All answers were scored by two independent raters that were blind to condition. The individual scores of the two raters were highly correlated, r = 0.98, *p* < 0.001. Conflicts between the two raters were solved by a third judge in order to get the final score for each participant.

#### Mental Effort

Mental effort was assessed with a translated and adapted version of the mental effort rating scale by Paas (1992) asking learners to rate mental effort (“How effortful did you find it to follow the content of the video?”) on a scale ranging from 1 (not effortful at all) to 9 (very effortful).

#### Evaluation of the Pauses

To investigate whether the attribution of the pauses affected participants' evaluation of the pauses, participants were asked to rate the disturbance caused by the pauses (“How annoying did you find the interruptions in the video?”) and the usefulness of the positions at which the pauses were positioned (“How useful were the positions where the video was interrupted?”). These ratings were collected on scales ranging from 1 (not at all) to 7 (very much).

#### Evaluation of the Video

To explore whether the attribution of the pauses also affected participants' overall evaluation of the video, participants were asked to answer questions about the video's interestingness (“How interesting did you find the content of the video?”), joy of learning (“How much did you enjoy learning from the video?”), the video's comprehensibility (“How well did you comprehend the content of the video?”), perceived difficulty (“How difficult did you find the content of the video?”), the quality of the explanation (“How high would you rate the quality of the explanations of the content?”), the intentions to use a similar video other topics in the future (“Would you like to see similar tutorials on other topics?”), and the suitability of videos to convey a foreign language (“How much are videos like this suitable for learning a foreign language?”). The participants' answers were recorded on scales ranging from 1 (not at all) to 7 (very).

#### Manipulation Check

Participants were asked whether they remembered the purported reason for the interruptions in the video in an open-ended question. The participants' answers were coded by one rater that was blind to condition according to a pre-defined coding scheme that classified the given reason into technical issues, didactical purpose, or other.

### Procedure

The study was conducted in a quiet room with up to four participants who took part in the experiment concurrently. Participants were seated at individual laptops with headphones and could not see each other's screens. The experiment was run in MediaLab 2016^3^. After giving informed consent, participants' prior knowledge and prior interest were assessed. Then, participants read the instructions, which were immediately followed by the Arabic language tutorial. After the tutorial, participants indicated mental effort before rating the video with regard to interestingness, joy of learning, comprehensibility, difficulty, future use intentions, and quality of the explanations. Then, participants indicated how disturbed they were by the pauses and whether they thought that the positions of the pauses were useful. This was followed by the question regarding the suitability of videos to learn a foreign language. Afterwards, participants filled in the knowledge test. The experiment ended with the manipulation check and the collection of demographic data such as age, gender, and mother language (German vs. Arabic vs. Other). Finally, participants were debriefed and financially compensated for their efforts. The procedure was approved by the local ethics committee.

## Results

### Prior Interest and Prior Knowledge

There were no group differences with regard to participants' prior interest in learning languages in general (*M* = 5.30, *SD* = 1.48), *F*(1, 62) = 0.01, *p* = 0.908, η^*2*^_*p*_ < 0.01, and the Arabic language in specific (*M* = 3.50, *SD* = 1.60), *F*(1, 62) = 0.10, *p* = 0.756, η^*2*^_*p*_ < 0.01. Further, the groups did not differ with regard to their ability to speak (*M* = 1.02, *SD* = 0.13), *F*(1, 62) = 0.78, *p* = 0.382, η^*2*^_*p*_ = 0.01, and understand the Arabic language (*M* = 1.03, *SD* = 0.18), *F*(1, 62) = 1.60, *p* = 0.211, η^*2*^_*p*_ = 0.03. Please refer to Table 1 for a full overview of the descriptive data.^4^

**Table 1:** Means (and standard deviation) for the variables assessed in the experiment.

	**Didactical pause**	**Technical issue**	**Overall**
Interest – General	5.32 (1.28)	5.28 (1.63)	5.30 (1.48)
Interest – Arabic	3.43 (1.48)	3.56 (1.72)	3.50 (1.60)
Prior Knowledge – Speak	1.00 (0.00)	1.03 (0.17)	1.02 (0.13)
Prior Knowledge – Understand	1.00 (0.00)	1.06 (0.23)	1.03 (0.18)
Knowledge score	12.34 (4.38)	12.51 (4.73)	12.44 (4.55)
Mental effort	5.71 (2.18)	4.89 (2.24)	5.25 (2.23)
Pause – Disturbing	3.39 (2.04)	3.83 (2.05)	3.64 (2.04)
Pause – Usefulness	4.86 (1.48)	4.28 (1.72)	4.53 (1.63)
Joy of learning	4.32 (1.49)	4.83 (1.50)	4.61 (1.51)
Interestingness	4.54 (1.20)	4.86 (1.50)	4.72 (1.37)
Quality of explanation	4.00 (1.56)	4.39 (1.32)	4.22 (1.43)
Comprehensibility	4.75 (1.62)	5.33 (1.66)	5.08 (1.66)
Perceived difficulty	4.14 (1.63)	3.64 (1.44)	3.86 (1.53)
Future use intentions	4.61 (2.01)	5.14 (1.57)	4.91 (1.78)
Suitability	4.00 (1.59)	4.00 (1.71)	4.00 (1.64)
N	28	36	64

### Knowledge Test and Mental Effort

An ANOVA revealed no significant differences between the two conditions (*M* = 12.44, *SD* = 4.55), *F*(1, 62) = 0.02, *p* = 0.880, η^*2*^_*p*_ < 0.01. The two conditions also did not differ with regard to participants' invested mental effort (*M* = 5.25, *SD* = 2.23), *F*(1, 62) = 2.19, *p* = 0.144, η^*2*^_*p*_ = 0.03.

### Evaluation of the Pauses

Regarding the evaluation of the pauses, there was no main effect of the attribution of the pauses for the perceived disturbance by the pauses (*M* = 3.64, *SD* = 2.04), *F*(1, 62) = 0.73, *p* = 0.396, η^*2*^_*p*_ = 0.01, or for the perceived usefulness of the positions of the pauses (*M* = 4.53, *SD* = 1.63), *F*(1, 62) = 2.02, *p* = 0.161, η^*2*^_*p*_ = 0.03.

### Evaluation of the Video

There were no main effects of the attribution of pauses for joy of learning (*M* = 4.61, *SD* = 1.51), *F*(1, 62) = 1.84, *p* = 0.180, η^*2*^_*p*_ = 0.03, interestingness (*M* = 4.72, *SD* = 1.37), *F*(1, 62) = 0.88, *p* = 0.351, η^*2*^_*p*_ = 0.01, perceived quality of the explanation (*M* = 4.22, *SD* = 1.43), *F*(1, 62) = 1.17, *p* = 0.284, η^*2*^_*p*_ = 0.02, comprehensibility (*M* = 5.08, *SD* = 1.66), *F*(1, 62) = 1.99, *p* = 0.164, η^*2*^_*p*_ = 0.03, perceived difficulty (*M* = 3.86, *SD* = 1.53), *F*(1, 62) = 1.73, *p* = 0.194, η^*2*^_*p*_ = 0.03, future use intentions (*M* = 4.91, *SD* = 1.78), *F*(1, 62) = 1.42, *p* = 0.239, η^*2*^_*p*_ = 0.02, and the participants' ratings whether videos were suitable for language learning, (*M* = 4.00, *SD* = 1.64), *F*(1, 62) < 0.01, *p* > 0.999, η^*2*^_*p*_ < 0.01.

### Correlations

Table 2 includes the correlations between all the assessed variables, except prior knowledge because there was hardly any variance regarding the prior knowledge scores. However, this section focusses on the associations between the main dependent variables and readers should remember that the correlational analyses were not pre-registered and should thus be considered exploratory. Whereas there were no associations between the learning outcomes and perceived disturbance, *r* = −0.09, *p* = 0.469, and learning outcomes and perceived usefulness, *r* = 0.19, *p* = 0.131, there was a significant correlation of perceived disturbance and perceived usefulness, *r* = −0.54, *p* < 0.001. In particular, if participants considered pauses to be placed at useful positions, they were less disturbed by their presence.

**Table 2:** Correlations between the different variables.

	**1**	**2**	**3**	**4**	**5**	**6**	**7**	**8**	**9**	**10**	**11**	**12**	**13**
1. Interest – General	-	0.34^**^	0.15	−0.13	−0.24	0.24	0.22	0.37^**^	0.21	0.14	−0.12	0.20	0.02
2. Interest – Arabic		-	0.12	−0.12	−0.20	0.07	0.44^***^	0.53^***^	0.08	0.31^*^	0.07	0.28^*^	0.21
3. Knowledge score			-	−0.34^**^	−0.09	0.19	0.29^*^	0.30^*^	0.25	0.21	−0.26^*^	0.14	0.13
4. Mental effort				-	0.12	−0.13	−0.25^*^	−0.26^*^	−0.29^*^	−0.60^***^	0.57^***^	−0.17	−0.29^*^
5. Pause – Disturbing					-	−0.54^***^	−0.09	−0.21	−0.13	−0.12	0.11	−0.09	−0.19
6. Pause – Usefulness						-	0.25^*^	0.22	0.37^**^	0.06	−0.26^*^	0.31^*^	0.34^**^
7. Joy of learning							-	0.66^***^	0.59^***^	0.53^***^	−0.15	0.67^***^	0.63^***^
8. Interestingness								-	0.46^***^	0.38^**^	−0.12	0.58^***^	0.36^**^
9. Quality of explanation									-	0.39^**^	−0.24	0.66^***^	0.59^***^
10. Comprehensibility										-	−0.38^**^	0.29^*^	0.44^***^
11. Perceived difficulty											-	−0.22	−0.29^*^
12. Future use intentions												-	0.61^***^
13. Suitability													-

*** *p < 0.001*.

** *p < 0.01*.

* *p < 0.05*.

## Discussion

This experiment investigated whether attributing the interruptions in a video either to a didactical purpose or to technical issues differentially affects the learning outcomes. In this regard, none of the pre-registered hypotheses was confirmed. In particular, pauses that were purportedly caused by technical issues did not have detrimental effects on learning (Hypothesis 1), were not considered to be more disturbing (Hypothesis 2), and were not considered to be placed at less useful positions (Hypothesis 3) than pauses that were purportedly introduced with a didactical purpose.

Despite these insignificant findings, readers should not use this experiment as an argument against investing into the technological infrastructure of schools, universities, or adult education centers in rural areas. Most importantly, the positions and the duration of the pauses in both conditions was exactly the same for methodological reasons. Thus, in order to ensure the credibility of the condition in that the pauses were purportedly introduced with a didactical purpose, this resulted in the limitation that the pauses never interrupted sentences and were never positioned within words. It is feasible to assume that technical issues such as buffering within words may have more detrimental effects on participants' evaluations than the effects observed in this experiment. Outside of the laboratory, such technical issues may be considered one of the major challenges for online learning and may thus decrease the participation in online courses (Song et al., 2004; Kay, 2012).

Objectively, the positions of the pauses were equally useful or disturbing across both conditions and the results of this experiment imply that the mere attribution of the pauses as either didactical or technical did not affect participants' evaluations of the pauses. On a positive note, technical issues were not more detrimental to learning than pauses that were introduced with a didactical purpose. However, on closer inspection, this finding also challenges the introduction of pauses into videos without taking into account whether the pauses are inserted at useful positions because pauses that were inserted with a didactical purpose were not considered to be more useful than technical interruptions *per se*. Given that this experiment revealed a negative association of the perceived usefulness of pauses and the perceived disturbance caused by the pauses, instructional designers may carefully want to check where to include pauses to avoid adverse effects of pauses.

With regard to the positioning of pauses, Merkt et al. (2018) systematically varied whether pauses in a video were positioned at meaningful structural breakpoints or at non-meaningful breakpoints. However, whereas Merkt et al. (2018) provide preliminary exploratory evidence that pauses at non-meaningful breakpoints may actually increase the learning outcomes; the authors did not assess the perceived usefulness or disturbance caused by the pauses. It is an interesting pathway for future research to investigate whether varying the position of pauses at meaningful or non-meaningful structural breakpoints together with the attribution of the pauses to technical issues affects learning.

Additionally, it is feasible to assume that a condition with unannounced pauses that catch viewers by surprise would be a closer match to real-world settings in that technical issues occur. However, it was decided not to include such a condition because, even though it is probable that most participants would attribute such interruptions to technical issues, some participants could still consider these pauses as part of the learning setting, the more so because the loading screen did not include any textual information hinting to technical issues. Therefore, such a condition was omitted from this experiment in order to have two groups that were internally homogeneous with regard to the attribution of pauses. Nevertheless, it is an interesting pathway for future research to include such a condition.

Further, the lack of a control condition that did not include any pauses could be seen as a limitation of the current experiment because the experiment does not provide any insights into whether the pauses, either with a didactical purpose or due to technical issues, positively affected the learning outcomes. However, it was decided against including such a condition because the experiment's focus was on investigating the consequences of attributing the pauses to different causes. More specifically, a condition without any pauses was not considered to be feasible because it would not have made much sense to collect data on perceived usefulness of the pauses or the disturbance caused by the pauses in such a condition. Nevertheless, future experiments that address related issues may want to include such a condition in order to establish the beneficial effects of pauses in the learning materials that were provided in the experiment.

Finally, the scores of the knowledge test imply that the contents of the video were rather difficult to learn. In particular, the participants only achieved about 23% of the maximum score in the knowledge test. This low score in the knowledge test may also be due to the participants' low levels of self-reported prior knowledge regarding the Arabic language, so that it is an interesting open question whether the findings of the current experiment generalize to more expert learners. Further, independent of condition, participants rated videos to be only moderately suitable for learning a foreign language (with an average rating of 4 on a scale ranging from 1 to 7). Even though we did not compare videos to other media such as static word lists, this data may shed some doubt on the suitability of videos for basic vocabulary learning, especially if learners have no prior knowledge regarding the foreign language. However, these findings should not be over-generalized to questioning the suitability of videos for language learning because language learning does not merely comprise the learning of basic vocabulary, which was the scope of the current experiment. In contrast, videos may also be used as authentic learning materials that allow students to listen to native speakers' pronunciation (Villegas Rogers and Medley, 1988; Colantoni et al., 2021). Thus, it may be useful to combine different representational formats in order to grasp the full potential of media for language learning.

In conclusion, the current experiment does not provide evidence that varying the attribution of pauses in videos to either a didactical purpose or to technical issues differentially affects the learning outcomes and the learners' evaluations of the usefulness or disturbance caused by the pauses. However, the discussion regarding the limitations of the experiment as well as the exploratory finding that the perceived disturbance caused by the pauses was negatively associated with the perceived usefulness of pauses may hopefully inspire future research diving deeper into both potentials and challenges of video-based learning.

## Data Availability Statement

The raw data supporting the conclusions of this article will be made available by the authors, without undue reservation.

## Ethics Statement

The study involving human participants was reviewed and approved by the Local Ethics Committee of the German Institute for Adult Education. The participants provided their written informed consent to participate in this study.

## Author Contributions

MM: conceptualization, data analysis, and writing.

## Conflict of Interest

The author declares that the research was conducted in the absence of any commercial or financial relationships that could be construed as a potential conflict of interest.

## Publisher's Note

All claims expressed in this article are solely those of the authors and do not necessarily represent those of their affiliated organizations, or those of the publisher, the editors and the reviewers. Any product that may be evaluated in this article, or claim that may be made by its manufacturer, is not guaranteed or endorsed by the publisher.

## Footnotes

^1^ https://osf.io/u2mcd/?view_only=98bdcbecfc6546a5b26f9ca94e40aa8a

^2^Please note that we had not pre-registered the participation in an Arabic language course as an exclusion criterion. Exclusion of this one participant did not affect the interpretation of any analyses.

^3^ https://www.empirisoft.com/download_old.aspx

^4^The manuscript reports the results of one-factorial ANOVAs. Following one of the reviewers' comments, I had also conducted ANCOVAs with both specific and general prior interest as covariates. Because including these covariates did not affect the interpretation of the findings, I decided to stick with ANOVAs to keep the results section short and simple.
